# Structure without semantics: no evidence for bonobo compositionality in Berthet et al. (2025)

**DOI:** 10.7717/peerj.21651

**Published:** 2026-08-21

**Authors:** Andreas Wartel, Johan Lind, Johan Lundin Kleberg, Claudio Tennie, Markus Jonsson, Anna Jon-And, Axel Ekström

**Affiliations:** 1 Centre for Cultural Evolution, Department of Psychology, Stockholm University, Stockholm, Sweden; 2 Department of Bird Ringing and Palynology, Swedish Museum of Natural History, Stockholm, Sweden; 3 Department of Psychology, Stockholm University, Stockholm, Sweden; 4 Faculty of Science, WG Early Prehistory and Quaternary Ecology, University of Tübingen, Tübingen, Germany; 5 The Lester E. Fisher Center for the Study and Conservation of Apes, Lincoln Park Zoo, Chicago, Illinois, United States; 6 EXC 3101 “HUMAN ORIGINS-Cluster of Excellence for Integrative Human Origins Studies”, University of Tübingen, Tübingen, Germany; 7 Department of Oncology-Pathology, Karolinska Institute, Stockholm, Sweden; 8 Department of Romance Studies and Classics, Stockholm University, Stockholm, Sweden; 9 Speech, Music & Hearing, KTH Royal Institute of Technology, Stockholm, Sweden; 10 Department of Language Studies, Umeå University, Umeå, Sweden

**Keywords:** Vocalization, Primates, Evolution of language, Statistics, Metascience

## Abstract

Understanding the evolutionary origins of combinatorial communication has motivated studies that analyse the semantic capacities of great ape vocal repertoires. A prominent example is the study by Berthet et al. (2025), which used Multiple Correspondence Analysis (MCA) on a large multidimensional dataset of bonobo calls and concluded that certain call combinations exhibit compositional meaning. We applied the published analytical pipeline to randomized datasets lacking any genuine association between call types and contextual features. Depending on implementation choices, the procedure identified significant relationships in 34.5–84.3% of simulations, suggesting substantial inflation of false-positive results. We therefore re-examined the statistical assumptions underlying the analysis itself. Because the MCA was constructed from repeated measures and uneven sampling across call types, its geometry reflects sampling frequencies and hierarchical dependencies in addition to any semantic structure. As such, any subsequent linear modelling of distances within this fixed space further violates assumptions of independence. We introduced a full permutation test in which a weighted MCA is recomputed under random reassignment of call-type labels, thereby generating a null distribution that respects the dependence structure of the data. Applying this procedure to the original dataset yielded a non-significant Monte Carlo *p*-value (*p* = 0.2597), indicating that the observed between-call-type separation does not exceed chance expectations. We argue that interpreting MCA distances as semantic relations requires theoretically motivated feature selection and dependence-aware inference, both of which are lacking in the original analysis. We conclude that Berthet et al. (2025) do not provide any statistically significant evidence for bonobo compositionality and highlight the broader limitations of relying on exploratory *post-hoc* statistical inference to identify semantic structure in animal communication.

## Introduction

The evolution of combinatorial spoken language—the ability to combine units into a near-infinite set—in the human lineage is a long-lived scientific puzzle (Hockett, 1960; Hurford, 2012; Lindblom, 1990; Townsend et al., 2018; Berthet et al., 2023; Jon-And et al., 2023). A recent trend in research on the evolution of language involves searching for “precursors” of such combinatorics in the calls of great apes—the closest living relatives of *Homo sapiens* (Leroux et al., 2023; Bortolato et al., 2023; Girard-Buttoz et al., 2022, 2025). The assumption underpinning many such works is that any capacity for combining vocal behaviors into sequences would shed light on the trajectory by which human ancestors acquired such an ability. One of the most recent in this line of studies was published in *Science* by Berthet, Surbeck & Townsend (2025) and targets the vocal behavior of wild bonobos (*Pan paniscus*).

Because learning mechanisms and capacities in humans and non-human animals are not identical, identifying appropriate features underpinning behavior is often difficult. This in turn motivates exploratory approaches to phenomena at hand. In this vein, Berthet et al. (2025) ambitiously set out to record the presence of a vast number of features of circumstances each time a bonobo uttered a call. Their data consists of 735 repeated measures of call types, each observation characterized by the presence or non-presence of 336 features of circumstances. A total of 170 observations are combined calls—defined as two single calls following each other within 2 s. The features included estimates of caller behavior at the time of emission and subsequent behaviors, the presence of external events, the subsequent behaviors of the visible audience, and changes in party composition. In total 30 individual bonobos were observed. Notably, the 19 call combinations that form the basis of the compositionality claims are represented by only 5–18 observations (for the least and most represented “combination”, respectively) from 3–12 individuals.

To handle such a large amount of features, Berthet et al. (2025) applied Multiple Correspondence Analysis (MCA), a dimension reducing technique that quantifies associations among categorical data. The coordinates of call observations within the MCA space can be viewed as a map of semantic meaning where points with similar feature profiles cluster together. Based on evidence derived from computing distances among call types within the MCA space, Berthet et al. (2025) argued that bonobos combine utterances in which the units do not contribute independent meaning, but modify each other. The authors interpret this as “non-trivial compositionality”. In this framework, semantic relationships are inferred from the relative positions of call-type barycenters within the MCA space, where each barycenter represents the average coordinate of all observations assigned to a given call type. Compositional call types are expected to occupy regions of the space that differ from those predicted by the meanings of their constituent calls alone.

According to this logic, for example, the “High-hoot_Low-hoot” should occupy a region separate from those occupied by the “high hoot” and “low hoot” call types, from which it is constituted. In the authors’ interpretation, such novel types of utterance combines meanings corresponding to “pay attention to me” (high hoot) and “I am excited” (low hoot) into “Pay attention to me, I am in distress”. Similarly, the “Peep_Whistle” merges the “I would like to…” *peep* and the “Let’s stay together” *whistle* into a signal for sensitive social contexts. If verified, observations such as those presented by Berthet et al. (2025) would indicate that combinations of calls extend the communicative repertoire of bonobos, thus providing new insights into their communicative capacities.

Our goal is not only to evaluate the conclusions of Berthet et al. (2025) but also to clarify the assumptions required for valid inference in high-dimensional analyses of animal communication. To this end, we develop a permutation-based alternative that respects the dependence structure of the data and discuss the broader challenges of interpreting semantic structure from highly exploratory feature sets. We conclude with implications for future research on animal communication and cognition.

## Reconstructing the space: a closer look at the Berthet data

Berthet et al.’s (2025) data consist of repeated measures of 26 bonobo call types—including seven single and 19 two-call combinations—where each call type is encoded by multiple categorical features. In their published article (Berthet, Surbeck & Townsend, 2025), statistical analyses of these data were performed by conducting a Multiple Correspondence Analysis (MCA), a multivariate statistical technique used to visualize and analyze the underlying structure and associations among nominal categorical variables by projecting both observations and variable categories into a low-dimensional Euclidean space (Greenacre, 2021). Call types appear as coordinates within this space and cluster together as a function of shared feature profiles.

Theoretically, the cloud of data points within the MCA space can be viewed as a map of semantic meaning. In order to test hypotheses about the relationship between call types, *e.g*., if two call types mean different things, Berthet et al. (2025) calculated linear models of the Euclidean distances between barycenters—weighted averages of each call type—within the MCA space. The following sections analyze the geometry of the MCA space and the statistical validity of Berthet et al.’s (2025) inferential procedure. We show that repeated-measures dependence, uneven sampling, and the treatment of distances within a fixed ordination space together create conditions under which apparent structure may arise even in the absence of meaningful call-type organization. We conclude by highlighting the importance of thorough statistical procedure in the search for language-like features in animal communication.

### Initial assessment

As an initial assessment of the robustness of Berthet et al.’s (2025) analytical pipeline, we applied the published procedure to randomized datasets lacking any genuine association between call types and contextual features. We generated 10,000 simulated datasets in which all feature variables were replaced by independent random binary values. Specifically, each feature was generated as an equal-probability Bernoulli process, such that every observation had a 50% probability of being coded “yes” and a 50% probability of being coded “no”, independently of call type and of all other features. Under these conditions, there is, by design, no non-random relationship between call types and feature profiles, and any statistically significant result therefore constitutes a false positive arising from the analytical procedure itself.

For each simulated dataset, we reproduced the portion of Berthet et al.’s (2025) analytical pipeline used to evaluate proposed compositional relationships among call types. Specifically, we recomputed the MCA, extracted the resulting call-type coordinates, calculated pairwise Euclidean distances among call types, and fitted the same distance-based linear model and subsequent pairwise comparisons used in the original study to determine whether combinations occupied a special position relative to their constituent calls. A simulation was scored as significant whenever at least one *post-hoc* comparison yielded p<0.05.

This procedure produced false positives at a marked rate. Under a nominal significance threshold of α=0.05, one would expect significance in approximately 5% of simulations. However, when all feature values were generated as independent Bernoulli variables, the analytical pipeline produced statistically significant results in 34.5% of simulations. We further examined the influence of the missing-data structure present in the original dataset by retaining NA values in their original locations while randomizing all observed feature values. This additional round of simulations, which thus more closely matched the conditions of the analysis reported by Berthet, Surbeck & Townsend (2025), produced a false-positive rate of 84.3%. That is, despite the absence of any underlying structure, the procedure employed by Berthet, Surbeck & Townsend (2025) frequently identifies significant relationships.

These findings indicate that the method can generate patterns that satisfy its own criteria for semantic interpretation even when no meaningful signal exists. In practical terms, the procedure frequently identifies combinations as occupying geometrically privileged positions relative to other call types despite the complete absence of any genuine association between call types and contextual features. The substantial increase in false-positive rates when the original missing-data structure was retained further suggests that stable geometric structure can arise from properties of the dataset itself, including patterns of missingness, rather than any semantic distinctions among call types.

### Overrepresentation of frequently sampled individuals

When constructing their MCA axes Berthet et al. (2025) did not take into account the repeated measures in their data. However, doing so is necessary because call types exert influence on the final MCA axes orientation in proportion to the number of observations of each call type. The consequence is that the MCA map starts reflecting what call types are *frequent* rather than *distinct*. In the following sections, we repeat the analysis procedure reported by Berthet et al. (2025) while controlling for these pitfalls.

Let ${n_{i}}$ denote the number of observations for call type i, with $N = \sum\nolimits_{i} {{n_{i}}}$. In an unweighted MCA, each observation contributes equally to the total inertia,

$I = \sum\limits_{i,j} d {({x_{ij}},g)^2},$

where ${x_{ij}}$ is the jth call of call type i, g is the grand centroid and $d{( \cdot , \cdot )^2}$ is the chi-square distance between the observation and g. If the aim is to map the *meaning* of call types, a correction for uneven sampling effort is necessary. This can be accomplished by a *weighted* MCA, where each observation is assigned a weight ${w_{ij}} = 1/({N_{{{\mathrm{types}}}}} \times {n_{i}})$, ensuring equal contribution per call type.

### Hierarchical structure of the data

Berthet et al.’s (2025) statistical method revolves around the analysis of the structure of the MCA space, in particular distances between barycenters. However, even after appropriately weighting an MCA (as was described above), distances within the MCA space cannot be considered independent and merely reflect profile distances in the raw data. Rather, the MCA space form a self-referential system where row coordinates are *barycenters* of category coordinates, and category coordinates are *barycenters* of all observations in which they appear. Changing any observation in the dataset changes the entire geometry of the MCA cloud. Parametric tests such as *t*-tests, are employed by Berthet, Surbeck & Townsend (2025), but are not conceptually consistent with the geometry of the MCA. Specifically, repeated measures follow a hierarchical model

${x_{ij}} = {\mu _{i}} + {\varepsilon _{ij}},$

where ${\mu _{i}}$ is the mean feature profile of call type i and ${\varepsilon _{ij}}$ represents within-type variation. MCA does not remove this dependence; instead, it incorporates it into the geometry of the space.

The coordinates of observations are obtained through a singular-value decomposition of the entire data matrix and therefore depend on all observations simultaneously. Consequently, any structure present in the repeated measurements—including their shared deviation from the true means ${\mu _{i}}$—is propagated into the coordinates of all points. Thus, the MCA barycenters are not fixed population means but estimates shaped by the same sampling noise that produced the individual observations. As a result, distances in MCA space reflect these dependencies and cannot be treated as statistically independent observations in subsequent parametric analyses.

### Shared endpoints

Regardless of the hierarchical structure of the data, Berthet et al. (2025) modeled distances between the MCA coordinates, treating each distance as an independent observation. However, because each coordinate participates in many distance pairs, observations such as d(A,B) and d(A,C) share a common endpoint A, yielding correlated residuals. Ordinary least squares assumes residual independence, rendering standard errors, t-values, and likelihood-ratio tests derived from such models unreliable and likely to inflate Type I error rates.

In summary, the statistical procedures applied by Berthet et al. (2025) operate on dependent quantities both because of shared endpoints and because the MCA coordinate system itself is data-dependent. Consequently, linear models applied to these distances do not satisfy the independence assumptions required for valid standard errors and significance tests.

### Use of “chance values”

Berthet et al. (2025) evaluate distances among call types by computing what they refer to as “chance values”, *i.e*., mean values resulting from repeatedly splitting each call type into random halves and measuring the distance between the artificial subsets. Effectively, the authors make the assumption that if another call type is found to lie outside this radius (as verified through a t-test), it can be concluded it is semantically distinct. However, this procedure measures the dispersion and clustering of call types *conditional* on the MCA embedding and does not test whether this embedding itself is caused by call types. Importantly, the geometry already contains the very structure that should be tested. The within-type distances come from the fixed MCA space and not independent, identically distributed samples from a common underlying distribution as assumed by a standard t-test. Therefore, “chance values” cannot function as baselines for significance testing.

### Supplementary variables do not correct dependencies

Berthet et al. (2025) claim to control for several variables, such as caller identity and number of calls, by including them as supplementary variables in the MCA. However, supplementary variables do not contribute to the construction of the MCA dimensions and are instead projected into an already computed space (Greenacre, 2021). Their inclusion therefore allows one to assess how these variables align with the resulting ordination but does not alter the geometry of the MCA itself. Consequently, supplementary variables cannot correct dependency structures arising from repeated measures or hierarchical organization in the underlying data.

### Permutation-based alternative

A more principled alternative to the method employed by Berthet et al. (2025) is to perform a *permutation test* in which the *weighted* MCA is recomputed under each random reassignment of call–type labels. This approach preserves the repeated-measures structure of the data and evaluates whether the entire MCA geometry—including axis orientation, singular values, and distances among barycenters—exhibits more between-type structure than expected by chance. In order to reassess the authors’ claims of “non-trivial compositionality” in bonobo calls, we applied this distinct approach to the dataset presented by Berthet, Surbeck & Townsend (2025).

For each permutation, call–type labels are randomly assigned to each row, MCA is then recomputed and the ratio of between–type to total inertia is recalculated:

$R = {{{I_{B}}} \over {{I_{T}}}},$

where ${I_{B}}$ is the between-type inertia derived from the permuted group barycenters and ${I_{T}}$ is the total inertia of that permuted MCA solution.

The null hypothesis is H_0_: *the observed association between call–type labels and feature profiles does not exceed chance expectations*.

Let

$\{ {R^{(1)}},{R^{(2)}}, \ldots ,{R^{(B)}}\}$

denote the values obtained from B independent permutations where the Monte Carlo *p*-value

$p={1 \over B}\sum\limits_{b = 1}^B {\bf{1}} \left( {{R^{(b)}} \ge {R_{{{\mathrm{obs}}}}}} \right),$

is the proportion of permuted datasets whose between–type structure is at least as large as the observed one.

When applied to Berthet et al.’s (2025) data with $B = 10{,}000$ permutations, we obtained

${R_{{{\mathrm{obs}}}}} = 0.121,\qquad p = 0.2597.$

Approximately 26% of permuted datasets exhibited equal or greater between-type separation than the empirical MCA. Thus, once the full MCA geometry and data dependence are accounted for, the clustering of call types does not exceed what would be expected under random assignment of call-type labels.

### Semantic interpretation and data collection

It was the intention of Berthet et al. (2025) to create a semantic space in which calls are represented as vectors in an MCA embedding. The dataset contains a large and diverse set of features ( n=336) describing acoustic, spatial, visual, and temporal aspects of call production and reception. However, feature selection that is not theoretically justified as indexing semantic meaning risks overwhelming the analysis with regularities unrelated to the construct of interest. In such circumstances, MCA will faithfully recover structure present in the data, but that structure need not be semantic. Our simulation results illustrate this concern. Specifically, the increase in false-positive rate when retaining the original pattern of missing values (*i.e*., that reported by Berthet et al. (2025)) suggests that the missing-data structure itself contains substantial regularity. Given such broad feature sets, stable multivariate structures can arise from several confounds unrelated to semantic distinctions—including observational constraints, sampling patterns, feature prevalence, or missing-data mechanisms. The fact that the observed between-call-type separation is compatible with chance under permutation is consistent with this interpretation.

### Summary

Our reanalysis used a permutation procedure in which call type was randomly assigned to each row in the data. For each permutation, a new MCA and the between-call types to total inertia ratio were calculated. In contrast to Berthet et al.’s (2025) use of linear models within a fixed MCA space, this procedure creates a null distribution that respects the dependencies among MCA coordinates under the hypothesis that call types contain no systematic information. With a Monte Carlo *p*-value of 0.2597 we conclude that the apparent separation of call types does not exceed what would be expected by chance.

## Discussion

MCA is an exploratory method that embeds categorical observations in a low-dimensional space whose geometry is entirely determined by the observed data. Distances in this space reflect not only systematic structure—such as repeated measurements, sampling imbalances, and correlations among features—but also the sampling noise present in the dataset. Because MCA has no mechanism for separating meaningful signal from noise, the map it produces is shaped by both—not only the properties a researcher hopes to interpret. Crucially, the MCA configuration is therefore not a fixed reference frame for statistical testing: any inferential model applied “within” this space reuses the same sampling noise and dependencies that created the embedding. Such reuse means that within-MCA tests cannot determine whether apparent clusters arise from a linguistic factor of interest or simply from the embedded noise and dependence structure. When we, in our full permutation test, fully respect sources of co-dependencies, the call type separations observed are compatible with chance. Overall, therefore, Berthet et al. (2025) do not present valid evidence for compositionality in the vocal system of bonobos. Other recent studies have pursued similar questions using smaller sets of contextual features and different inferential frameworks (*e.g*., Girard-Buttoz et al., 2025), thus avoiding some of the specific inferential issues discussed here. However, as with any exploratory analysis of complex observational data, conclusions remain contingent on the assumptions embedded in the chosen analytical framework.

As research on animal communication increasingly seeks parallels to human language, the burden is rising on statistical and theoretical rigor. Exploratory methods risk creating the appearance of linguistic “precursors” where none exist, potentially misleading future work in the field. Compositionality is a linguistically precise concept, requiring not only distinguishable units but also rule-governed combination that yields predictable, interpretable meaning. Demonstrating such a system in wild apes demands analytical methods capable of separating genuine semantic structure from the substantial noise inherent to field data. Only by integrating methodological precision with clear linguistic criteria can the field hope to identify genuine signatures of compositionality—or to understand why such signatures may be absent—in our closest living relatives.

Given that the methods, as applied by Berthet, Surbeck & Townsend (2025) are biased by the frequency of data in a dataset, subsequent studies using the same methodology would almost certainly also “find” evidence for novel behaviors in animal communication. Problems resulting from inaccurate statistical procedures may be compounded by various systematic problems outside the integrity of scientific work itself (Benjamin et al., 2018), including the documented resistance from top journals to publishing critiques of published articles (Brainard, 2022)^1^ 1Our observations were originally submitted as a comment on the original publication in *Science* (Berthet, Surbeck & Townsend, 2025), but the submission was desk rejected by the handling editor without peer review.—despite an equally documented high prevalence of unreliable findings (Fang & Casadevall, 2011; Nature, 2014). As a consequence, unreplicable or unreliable findings may become entrenched in relevant literatures; the field of animal cognition and communication research is not exempt from this danger (Mizuno et al., 2025). To counteract this trend, it is necessary that sufficiently powerful statistical procedures are developed, and that implications of their use and misuse are fully understood.

## Additional Information and Declarations

### Data Availability

The following information was supplied regarding data availability:

The code and data are available at GitHub and Zenodo:

- https://github.com/AndreasWartel/Bonobo-MCA/tree/main.

- AndreasWartel. (2026). AndreasWartel/Bonobo-MCA: v.1.0.0 (Version v.1.0.0) [Computer software]. Zenodo. https://doi.org/10.5281/zenodo.21370080.
